# Associations between Maternal and Offspring Hair Cortisol Concentrations and Child Behavioral Symptoms in Mother-Child Pairs with Perinatal Mental Disorders

**DOI:** 10.3390/children9060810

**Published:** 2022-05-31

**Authors:** Anna Agapaki, Fenia Papagianni, Dimitra Metallinou, Eleni Valavani, Aimilia Mantzou, Stamatia Kanelli, Makarios Eleftheriades, Areti C. Spyropoulou, Ioannis Zervas, George P. Chrousos, Panagiota Pervanidou

**Affiliations:** 1 Unit of Developmental and Behavioral Pediatrics, First Department of Pediatrics, School of Medicine, National and Kapodistrian University of Athens, Aghia Sophia Children’s Hospital, 11527 Athens, Greece; anna.agapaki@gmail.com (A.A.); f.papagianni@yahoo.gr (F.P.); elvalav@yahoo.com (E.V.); amantzou@med.uoa.gr (A.M.); chrousge@med.uoa.gr (G.P.C.); ppervanid@med.uoa.gr (P.P.); 2 Department of Midwifery, University of West Attica, 12243 Athens, Greece; 3 Women’s Mental Health Clinic, First Department of Psychiatry, School of Medicine, National and Kapodistrian University of Athens, Eginition Hospital, 11528 Athens, Greece; kanellitina@yahoo.com (S.K.); arspyrop@yahoo.com (A.C.S.); izervas@med.uoa.gr (I.Z.); 4 Second Department of Obstetrics and Gynaecology, Aretaieio Hospital, National and Kapodistrian University of Athens, 11528 Athens, Greece; makarios@hotmail.co.uk; 5 University Research Institute of Maternal and Child Health and Precision Medicine and UNESCO Chair on Adolescent Health Care, National and Kapodistrian University of Athens, 11527 Athens, Greece

**Keywords:** perinatal mental disorders, hair cortisol, maternal stress, developmental and behavioral problems

## Abstract

Maternal perinatal mental disorders (PMD) are associated with developmental and behavioral problems in children, probably mediated by the programming of the hypothalamic–pituitary–adrenal (HPA) axis. Increased cortisol concentrations during the antenatal and perinatal periods have been related to long-term effects on children’s behavior and stress response. We aimed to investigate the association of hair cortisol concentrations (HCC) between mothers, with (n = 16) and without PMD (n = 30), and their children, aged between 18 and 48 months. Participants were evaluated with a clinical interview and questionnaires for the Depression Anxiety Stress Scale and the Child Behavior Checklist for ages 1½–5. Maternal and child HCCs were compared between the two groups. Children of the PMD group had increased symptoms of attention deficit hyperactivity disorder. A positive linear association between maternal and child HCC was observed only in the total sample of mother–child dyads and the control group. In the PMD group, children’s HCCs were significantly associated with child anxiety/depression symptoms. Aggressive behavior and oppositional/defiant problems correlated significantly with children’s own HCCs, and their mother’s too. These findings suggest that a chronic dysregulation of maternal and child HPA axis and their associations in the PMD dyads may underlie the linkage among prolonged maternal stress, child behavioral/emotional problems and stress responses.

## 1. Introduction

Depressive and anxiety disorders in general, are associated with dysregulation of the hypothalamic–pituitary–adrenal (HPA) axis and dysfunction of the autonomic nervous system, with the peripheral biomarkers of both axes being altered in such disorders. Similarly, perinatal mental disorders (PMD) are linked to the dysregulation of the maternal HPA axis [1], which may often be prolonged [2].

In clinical research, the measurement of cortisol concentrations in the periphery is a well-established method to assess the activity of the HPA axis. Salivary cortisol in a diurnal sample is considered to evaluate stress on that particular day, whereas accumulative hair cortisol is defined as an indicator of chronic stress exposure [3]. In human studies, the presence of a maternal PMD may have long-term effects on a child’s response to stress [4]. Maternal prenatal stress has been closely related to elevated basal cortisol levels in children and dysregulation of the HPA axis [5] via fetal programming. 

Limited studies have investigated the association of hair cortisol concentrations (HCC) between the mother–child dyad [6,7,8]. Previous research has shown that the HCC of mothers and children were highly correlated, suggesting a heritable trait or maternal calibration of the child’s HPA axis activity [7]. However, this correlation is not always observed [9]. Although the etiology of contradictory results concerning early childhood is not yet specified, it must be taken into consideration that the regulation of the HPA axis during that period is not well established, and that the population, methodological tools and age groups may vary between studies [10]. 

As far as the impact of PMDs on children’s behavior is concerned, longitudinal studies have shown that children whose mothers were diagnosed with PMD demonstrated emotional and behavioral problems [11]. Maternal PMDs have been associated with both internalizing (e.g., depression, anxiety) and externalizing problems (e.g., hyperactivity, aggression or delinquency), with regard to early childhood [11]. The relationship between regulation of the HPA axis and behavioral problems in children has been also studied repeatedly, suggesting pathways through which cortisol may influence children’s behavior long-term [12]. However, hair cortisol has not yet been extensively examined in association with behavioral and emotional problems in children and findings are still inconsistent [13]. 

In this case-control study, we aimed to investigate the association of HCC between mothers, with and without PMD, and their children, aged between 18 and 48 months. Furthermore, we sought to determine the prevalence of behavioral and emotional disorders in the child population of both groups and to examine the linkage between these disorders and hair cortisol of both the children and their mothers. We hypothesized a positive correlation between maternal and child hair cortisol in the control group and significant associations between mothers with a PMD diagnosis and their children’s behavioral problems. 

## 2. Materials and Methods

### 2.1. Study Design and Participants

This is a cross-sectional study, which is part of a broader research protocol on stress system activity in children and adolescents, and their parents/caregivers. The study conformed to the standards of the latest version of the Declaration of Helsinki, and was approved by the Ethics Committee of the ‘Aghia Sophia’ Children’s Hospital in Athens, Greece (protocol code: 29012/21 December 2015). 

The first 50 mother–child dyads that met the inclusion criteria participated in the survey, with a sex ratio of 1:1. The final study population consisted of 92 individuals (mothers and their children). In detail, the case group was constituted of 16 mothers with a history of PMD and their children, and the control group included 30 mother–child dyads. The chronological age of all children ranged from 18 to 48 months.

The inclusion criteria were: (i) Maternal age between 18 and 45 years old, (ii) Mothers literate of the Greek language, and (iii) Mothers with a perinatal period that coincided with the aforementioned recruitment period. Exclusion criteria were: (i) A multiple pregnancy, (ii) Prematurity and/or intrauterine growth restriction (IUGR), (iii) Maternal pathological conditions during pregnancy, such as preeclampsia or diabetes mellitus, (iv) Maternal chronic diseases and conditions, (v) Conception after in vitro fertilization (IVF), (vi) Surgery during pregnancy, (vii) Maternal use of illicit/addictive substances during the perinatal period, (viii) Family history of neurodevelopmental disorders, (ix) Abnormalities of the uterus and/or cervix, (x) Glucocorticoid intake, (xii) Perinatal complications, such as hypoxia, respiratory problems or seizures [14], and finally, (xiii) Children with cerebral palsy, chromosomal abnormalities, genetic disorders or a known developmental disability. 

### 2.2. Procedure of Recruitment

Eligible mothers were adequately explained and informed about the purpose and the nature of the study by a preassigned staff nurse from the clinic. Mothers of the control group were enrolled in the study after a written announcement to public kindergartens in the same region as the case group. Recruitment was in agreement with all ethical considerations (voluntariness, confidentiality etc.) and sufficient time was offered to all prospective participants to consider whether or not they are interested in proceeding to the written informed consent. No direct personal benefits were provided to research participants.

### 2.3. Clinical Evaluation and Diagnoses 

Mothers with PMDs were evaluated at the ‘Women’s Mental Health and Perinatal Psychiatry Clinic’ of the First Department of Psychiatry, School of Medicine, National and Kapodistrian University of Athens, Greece. Control mothers were evaluated through a retrospective clinical interview, focused on the perinatal period, which was conducted by a clinical psychologist in order to exclude those with perinatal symptoms of stress, depression or anxiety or any other mental illness, and besides, those who did not fulfill the inclusion criteria. 

#### 2.3.1. Maternal Measures

A questionnaire designed by the research team was distributed to both groups so as to acquire maternal demographic, occupational and individual characteristics including age, education, occupation and perinatal data. 

Previous maternal psychiatric diagnosis of PMD relied on the Diagnostic and Statistical Manual of Mental Disorders, Fourth Edition, Text Revision (DSM–IV-TR) which was given by certified psychiatrists who specialized in PMD, using the Greek version of the semi-structured clinical interview, i.e., Structured Clinical Interview for DSM-III-R (SCID-NP) [15]. All mothers of the case group were recruited from the same clinic with the same diagnostic methods being used.

A semi-structured clinical interview, including the perinatal psychiatric history, was performed by a certified clinical psychologist in order to screen mothers for the control group. Depressive symptoms and/or signs of anxiety and/or stress during the perinatal period were examined retrospectively. Those who reported moderate or severe symptoms of depression, anxiety or stress were excluded from the study.

Current maternal emotional state was assessed through: (a) A semi-structured clinical interview performed by the clinical psychologist and (b) The Greek version [16] of the Depression Anxiety Stress Scale (DASS-42) [17]. The DASS is a 42-item self-reporting instrument designed to measure the three related negative emotional states of depression, anxiety and tension/stress. It is a set of three scales, each of which contains 14 items divided into subscales of 2–5 items with similar content. Mothers rated the extent to which they had experienced each state over the past week through a Likert scale with gradations between 0 and 3. The total scale of the DASS-42 has a Cronbach alpha of 0.968 and coefficient alphas for each scale are α = 0.941 for depression, α = 0.906 for anxiety and α = 0.942 for stress [16].

#### 2.3.2. Children’s Measures

The developmental and behavioral history and assessment of all children, as well as the clinical (pediatric) examinations, were all performed by a certified developmental pediatrician. A full physical examination was conducted and current weight and height were measured. The Gesell Developmental Schedules (Revised) were used as a part of the developmental evaluation [18]. Speech and language of children were assessed by a certified speech and language therapist with the use of the Preschool Language Scales, Fifth Edition [19].

The Greek version of the Child Behavior Checklist for preschool children (CBCL/1½–5) [20] was completed by both the parents and teachers of each child. The CBCL is a widely used instrument in clinical practice and research to assess a broad range of behavioral and emotional problems. It provides information on internalizing problems, suggesting problems of emotional reactivity, anxiety/depression, somatic complaints and withdrawal, as well as of externalizing problems, suggesting problems of attention and aggression. The total score (T-score) is calculated by adding the scores of all the problem items. Furthermore, the CBCL includes a set of “DSM-oriented” scales, examining: (i) Affective problems, (ii) Anxiety problems, (iii) Pervasive developmental problems, (iv) Attention Deficit/Hyperactivity Problems, and (v) Oppositional/Defiant Problems. Cronbach’s alpha for each scale in the Greek version ranges from 0.52 to 0.87. Cronbach’s alpha for internalizing and externalizing problems is 0.82 and 0.89, respectively. The data were inserted into the Achenbach System of Empirically Based Assessment (ASEBA) application [21] and T-scores of symptoms, based on Greek norms, were calculated in order to obtain a clinical interpretation of the results.

#### 2.3.3. Hair Sample Collection and Analysis

Hair was cut as close as possible to the scalp from the posterior vertex of the head from mothers and their children. The samples were kept at room temperature until they were analyzed. From each hair sample, 3 cm was used for the analysis. Hair was placed in tubes (Precellys Lysing Kits, Bertin Technologies, Paris, France), grinded and pulverized at 5000 rounds per minute via a homogenizer (Minilys, Bertin Technologies, Paris, France). Following this, 1 mL of methanol was added to each tube at room temperature and stirred for 16 h. The samples were centrifuged (Biofuge 13, Heraeus Instruments, Athens, Greece) and the extracts were transferred to a glass tube and left at room temperature in order to evaporate until they dried. Afterwards, they were reconstituted with phosphate buffered saline (100 μL) (pH 8.0) and placed in a vortex for 1.5 min. Before analysis, the samples were placed in a vortex once more. Finally, to estimate cortisol levels, the automatic electrochemiluminescence immunoassay “Cortisol II” was used in the automatic analyzer Cobas e411-Roche Diagnostics (GmbH, Mannheim, Germany). The detection threshold, as indicated from the manufacturer’s instructions, was set at 0.054 μg/dL. Hair cortisol was then calculated and expressed in pg/mg of hair.

### 2.4. Statistical Analysis 

The mean, standard deviation (SD), median and interquartile range were used to present quantitative variables, whereas absolute (N) and relative (%) frequencies were used to present categorical variables. Comparison of proportions was performed with the Pearson’s chi-squared test or the Fisher’s exact test, where appropriate. Comparison of quantitative variables between the groups was performed with the Student’s t-test or the non-parametric Mann–Whitney U test. Pearson’s correlation coefficient (r) was used to estimate the relationship between quantitative variables. The correlation is considered low, moderate or high when the correlation coefficient (r) ranges are (0.1–0.3), (0.31–0.5) and >0.5, respectively. Significance levels of bilaterally and statistical significance was set at 0.05. All statistical analyses were performed using the SPSS v.19.0 package.

## 3. Results

The study included 16 mothers with PMD and their children, and 30 age-matched control mothers and their children. Maternal and child characteristics are presented in Table 1 and Table 2, respectively.

### 3.1. Behavioral and Emotional Data

The developmental assessment did not reveal significant differences in clinical diagnoses of the children between the two groups. All children were of typical development; however, low–normal levels were noted in expressive language in 50% of children from mothers with PMD vs. the 33.3% of children from the control group. Similarly, low–normal levels in the social–emotional domain were noted in the 18.8% of children from mothers with PMD vs. 13.3% of children from the control group. The behavioral assessment was performed with the use of the standardized CBCL questionnaires. Mothers from the PMD group scored significantly higher attention deficit hyperactivity disorder (ADHD) symptoms in their children (*p =* 0.035) compared to the control children. No significant differences were observed in the rest of the symptom scales. 

Regarding the evaluation of the current maternal psychological status, the DASS-42 questionnaire revealed higher levels of symptoms of depression, anxiety and stress in case mothers, in contrast to the control mothers. The scores of DASS-42 scales of depression, anxiety and stress derived from the control group, were 4.3 ± 5.3, 4.1 ± 4.8 and 9.8 ± 5.7 whereas, for PMD mothers, they were 7.1 ± 9.3, 6.8 ± 6 and 11.9 ± 7.5, respectively. Nevertheless, these differences were not significant (*p* = 0.228 for depression symptoms, *p* = 0.123 for anxiety symptoms and *p* = 0.305 for stress symptoms). 

The DASS-42 and CBCL scores correlated positively at several points. In the entire sample, maternal depressive symptoms correlated with their child’s emotionally reactive scores (*r* = 0.33, *p* = 0.037), withdrawn scores (*r* = 0.40, *p* = 0.009), internalizing problems scores (*r* = 0.44, *p* = 0.004), externalizing problems scores (*r* = 0.36, *p* = 0.020), total problems scores (*r* = 0.43, *p* = 0.005), affective problems scale (*r* = 0.32, *p* = 0.043) and pervasive developmental problems scale (*r* = 0.45, *p* = 0.003). Maternal anxiety symptoms correlated with the child’s attention problem scores (*r* = 0.45, *p* = 0.004), externalizing problems scale (*r* = 0.36, *p* = 0.023), total problems scores (*r* = 0.37, *p* = 0.020), pervasive developmental problems scale (*r* = 0.36, *p* = 0.021) and attention deficit/hyperactivity problem scores (r = 0.41, *p* = 0.008). Maternal stress symptoms correlated with their child’s internalizing scores (r = 0.38, *p* = 0.015), total problems scores (*r* = 0.42, *p* = 0.006), and pervasive developmental problems scale (*r* = 0.41, *p* = 0.008). 

Furthermore in the control group, maternal depressive symptoms correlated with their child’s emotionally reactive scores (*r =* 0.47, *p* = 0.017), withdrawn scores (*r =* 0.41, *p* = 0.044), externalizing problems scores (*r =* 0.43, *p* = 0.028), total problems scores (*r =* 0.43, *p* = 0.031), affective problems scale (*r =* 0.44, *p* = 0.043) and pervasive developmental problems scale (*r =* 0.44, *p =* 0.027). Maternal anxiety symptoms correlated with their child’s emotionally reactive scores (*r =* 0.46, *p =* 0.024), attention problem scores (*r =* 0.58, *p =* 0.003), internalizing problems scale (*r =* 0.44, *p =* 0.030), externalizing problems scale (*r =* 0.48, *p =* 0.017), total problems (*r =* 0.52, *p =* 0.009), affective problems scales (*r =* 0.52, *p =* 0.009), pervasive developmental problems scale (*r =* 0.49, *p =* 0.016) and oppositional defiant problem scores (*r =* 0.42, *p =* 0.041). Maternal stress symptoms correlated with the child’s emotionally reactive scores (*r =* 0.41, *p =* 0.041), total problems scores (*r =* 0.41, *p =* 0.039), pervasive developmental problems scale (*r =* 0.44, *p =* 0.029) and attention deficit /hyperactivity problems (*r =* 0.41, *p =* 0.041).

Finally, in the PMD group, maternal depressive symptoms correlated with their child’s somatic complaints scores (*r =* 0.62, *p =* 0.011), internalizing scores (*r =* 0.54, *p =* 0.032), pervasive developmental problems scale (*r =* 0.52, *p =* 0.040). Maternal anxiety and stress symptoms showed no significant correlations with any scale. 

### 3.2. Maternal and Child Hair Cortisol Concentrations

**Figure 1:**
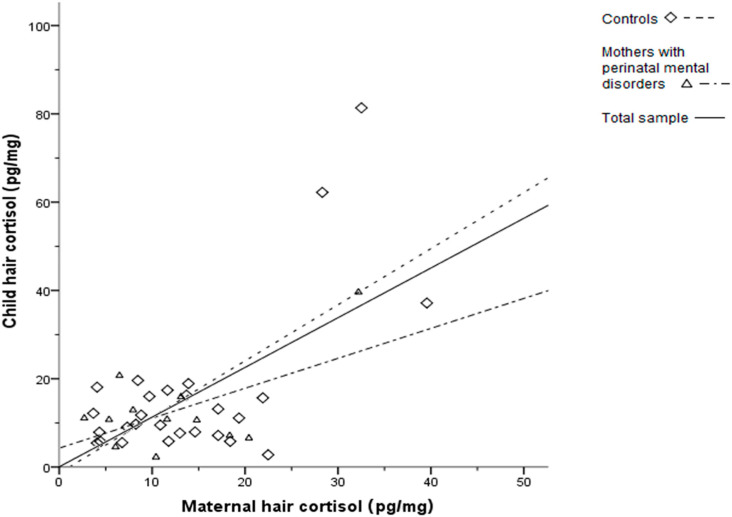
Relationship between maternal and child hair cortisol. Child hair cortisol (ordinate) is increased along with maternal hair cortisol (abscissa), for the total sample and each group separately; Control group, N = 30; PMD group, N = 16; PMD: perinatal mental disorders.

Mothers with PMD had lower HCC (11.8 ± 8.2 pg/mg) compared to the control mothers (13.9 ± 9 pg/mg), although that difference was not significant (*p =* 0.471). Similarly, children of the PMD group had lower HCC (12 ± 9.7 pg/mg) in comparison with the control children (15.9 ± 17.4 pg/mg) and that difference was not significant too (*p =* 0.455). In the entire sample, maternal HCC correlated positively with the hair cortisol of their children (*r =* 0.63, *p <* 0.001). The same positive correlation was also found in the control group (*r =* 0.63, *p <* 0.001) but was not demonstrated in the PMD group (*r =* 0.57, *p =* 0.05). Figure 1 illustrates the positive linear relationship between maternal and child hair cortisol for the total sample and each studied group separately.

### 3.3. Maternal and Children’s Hair Cortisol Concentrations and CBCL Scales

The scores summed from mothers in the CBCL scale, were analyzed separately for each group. Neither in the entire sample nor in the control group, did HCC of mothers or children show significant correlations with any of the CBCL scales. As for the PMD mother–child dyads, the T-scores of the “anxious/depressed” scale correlated positively and significantly with children’s HCC, whereas T-scores of the “aggressive behavior” and the “oppositional defiant problems” correlated positively and significantly with both maternal and child HCC. The results are presented broadly in Table 3.

## 4. Discussion

This study demonstrated no differences in clinical developmental diagnoses among children of mothers with or without PMD. Nonetheless, ADHD symptoms, as reported by mothers, were increased in children whose mothers were diagnosed with PMD. Current maternal symptoms of depression, anxiety and stress correlated with a variety of behavioral and emotional symptoms and problems in children, in the entire and the control group, but few correlations were noted in the PMD group.

In the present study, children of both groups were of typical development and showed no differences with clinical importance. These findings can be explained firstly by the stringent inclusion criteria which were applied in the study (e.g., children with prematurity, IUGR and perinatal complications were excluded) and secondly, by the fact that mothers with PMD had systematic psychological support from a healthcare professional and their family as well. A possible additional explanation of that finding could also be the young age of the participating children (18–48 months old). In this early developmental stage, only moderate and severe neurodevelopmental disorders can be diagnosed, whereas, mild developmental disorders, learning and mental health problems typically appear in the later developmental stages. However, on a subclinical level, children of the PMD group more often had mild expressive language and social problems compared to the control group. Our results are in good agreement with previous studies, which have demonstrated that children exposed to high levels of prenatal stress have lower cognitive, intellectual and linguistic abilities [22,23]. Moreover, parenting is a key factor able to clarify some of the difficulties that children from mothers with PMD face [11]. For example, the low percentage of breastfeeding among mothers with PMD might have a strong implication in the results of our study; this evidence is also supported by previous authors [24,25]. Besides, it should not be neglected that 50% of mothers with PMD were receiving appropriate medication. In a large prospective study by Skurtveit et al. [26], prolonged use of selective serotonin reuptake inhibitors (SSRIs) during pregnancy was significantly correlated with speech delay in 3-year-old children, regardless of the occurrence of maternal anxiety/depressive symptomatology. In our study, the medical history of PMD mothers featured that their concerns of possible exposure of their child to psychotropic ingredients, especially through breastfeeding, motivated them to continue to be monitored by a healthcare professional, a fact that probably reduced the potential risks of recurrence during the postpartum period.

Considering the possible correlation between perinatal exposure of children to maternal PMDs through cortisol levels and child behavioral disorders, it was found that PMD mothers evaluated attention deficit/hyperactivity problems with higher scores in the CBCL scale. Previous studies have shown that maternal emotional disorders, especially during the postpartum period, have negative effects on children, such as attention problems [27]. Van Batenburg-Eddes et al. [28] reported a strong association between antenatal anxiety disorders and childhood disorders. Furthermore, Velders et al. [14], using the CBCL scale, found that children’s behavioral problems correlated highly with maternal depression during pregnancy. Additionally, studies in monkeys have demonstrated that maternal stress during pregnancy led to smaller periods of their offspring’s attention [29]. The above is in line with the findings of our research, while it stresses out the impact of maternal stress and mental disorders during pregnancy on the duration of a child’s attention and hyperactivity.

As far as HCCs are concerned, the correlation between maternal and child hair cortisol was significantly positive, both in the total sample and per group, though it was weaker in the PMD group. Therefore, the higher the maternal hair cortisol levels, the higher they were in the children. A limited number of studies have shown high positive correlations between maternal hair cortisol and that of their children as a chronic stress marker [7,10,30]. Yet, there are controversial observations about hair cortisol in infancy and early childhood [9], reflecting the ongoing modulation of the HPA axis function and, therefore, findings vary among the different cultures, methodological tools and age groups that each study uses [10]. It is important to note that only few recent studies have examined the deviation of such correlation in a PMD group [31,32].

The observation that mothers who manifest stressful behaviors are less able to help their children with the regulation of the HPA axis [33], could explain this attuned association of HCC. Moreover, mothers and children share the same environment and therefore may be exposed to similar stressors, resulting in similar cortisol levels. A third mechanism that may be involved is the process of fetal programming and the effects of the family environment on the child. It has been already shown that prenatal maternal HCC can predict the child’s hair cortisol levels from their first to third year of life [7]. We report no differences in hair cortisol between the two studied groups. This result could be favored by the restorative operations of PMD mothers (i.e., psychological support, medication) which could possibly affect their children at the same time.

With regard to the scores of the DASS-42 scale dimensions, they did not differ between the two studied groups, possibly due to the small sample size. The self-reported nature of this questionnaire, which explores the current maternal psychological condition, might have distorted the scores. In addition, correlations between maternal and child hair cortisol and the dimensions of the DASS-42 scale, was not significant. These findings are in agreement with other studies that have highlighted the absence of a correlation between child hair cortisol and maternal perceived stress or depressive symptoms [9,34]. However, they are in contrast with the results of Palmer et al. [13] who found that high levels of hair cortisol have a high correlation with parental stress and depressive symptoms of the mother.

Examining the relationship between maternal and child hair cortisol and the dimensions of the CBCL scale in the PMD group, there was a significant positive correlation between maternal hair cortisol and the dimensions of “aggressive behavior” and “oppositional defiant problems”. Moreover, higher hair cortisol levels of children from PMD mothers were associated with higher values in “anxious/depressed”, “aggressive behavior” and “oppositional defiant problems”. This finding emerging from our study, that cortisol is associated with child behavioral problems, suggests that maternal prenatal stress can actually influence the temperament of the child. In particular, high maternal levels of cortisol during pregnancy appear to alter the neurotransmitter activity of the fetus, especially during the period of brain development. Alternations in the activity of corticotropin-releasing hormone (CRH) and other neurotransmitters that underlie, increase the anxiety and depressive behavior of the child, indicating that maternal stress hormones may be responsible for the programming of the relevant neural systems of the child [35]. In our study, children whose mothers faced PMD were not found to have internalized or externalized problems. This result substantiates the previous observation that maternal perinatal depression is not a determining risk factor for developing behavioral disorders in children [36].

## 5. Limitations

We are aware that our research has some limitations. A main limitation of the present study is the small sample size due to the child age limits and poor maternal response to participate in both sample groups. However, given the fact that mothers with perinatal mental disorders are a ‘hard-to-reach’ population, meaning that they constitute a sub-group of the population that is difficult to reach or involve in research, we believe that these preliminary findings, which only allow for an initial exploration and cannot be generalized in the population, do contribute to the scientific field as per previous studies [31]. The difficulty in interpreting the above results is increased considering the heterogeneity of maternal PMDs, as well as the severity of their symptoms and how they affect the child’s development and behavior.

Also, as mentioned above, the motivation of mothers without PMD for participating in this study, was to assess their child’s development and overall functioning, which may indicate strong concern for their child’s development and upbringing. However, the same could be asserted for PMD mothers, as most of them were aware of medication aggravating effects on their fetus during pregnancy, and we assume that their worry about their child’s development was elevated.

Additional study’s limitations include the retrospective nature of the medical history of PMD mothers. As a result, there is a recall bias about specific information regarding their perinatal emotional state, as well as the developmental history of their children.

Finally, the technique of hair acquisitioning and analyzing satisfied all requirements. Nevertheless, it was not possible to eliminate unstable factors which affect cortisol hair levels such as dietary patterns, shampooing frequency, dyeing hair and the usage of conditioners and creams with steroids [37,38].

## 6. Conclusions

There are several possible explanations of the modified regulation of feedback tracks at the HPA axis and its hyperactivity: (1) reduction of receptor regulation in the hippocampus, hypothalamus, pituitary or adrenal glands, (2) reduced receptor sensitivity at any of these levels, and (3) alteration of CRH levels or plasma binding protein levels or their affinity. Although the mechanisms that can modify feedback on the HPA axis after prenatal stress have not been fully investigated, there are at least two indications: reduction in hippocampal corticosteroid receptors and higher CRH levels in the amygdala of children exposed to endometrial stress.

It is well known that elevated maternal cortisol concentrations during pregnancy affect fetal brain development. Cortisol crosses the blood–brain barrier and affects the limbic system of the brain, as amygdala, which is involved in the regulation of emotional behaviors. Increased CRH levels, induced by glucocorticoids, have permanent effects, especially on the amygdala development and function, leading to anxiety problems in children. It should be stressed that the professional support through medication and psychological reinforcement of PMD mothers during the antepartum and postpartum periods, could efficiently assist the physiological early development and behavior of children.

## Figures and Tables

**Table 1:** Maternal characteristics (N = 46).

	Control Group (Ν = 30)	Mothers with Perinatal Mental Disorders (Ν = 16)	*p*
Age, mean (SD)	36.3 (3.6)	36.6 (4.1)	0.801 ^1^
ΒΜΙ, mean (SD)	22.1 (4.1)	24 (4.5)	0.200 ^1^
Education, N (%)			0.020 ^2^
Highschool	9 (32.2)	8 (53.4)	
University	7 (25.0)	6 (40.0)	
MSc/PhD	12 (42.9)	1 (6.7)	
Employment status, N (%)			
Employed	27 (89.3)	9 (56.2)	
Unemployed	3 (10.7)	7 (43.8)	
Diagnosis, N (%)			
Mood disorders with psychotic features		3 (18.8)	
Mood disorders without psychotic features		1 (6.3)	
Depression		9 (56.3)	
Anxiety disorder		3 (18.8)	
History of other mental disease, N (%)	1 (3.3)	13 (81.3)	<0.001 ^2,^*
Self-reported stress symptoms during antenatal period, N (%)	12 (40.0)	12 (75.0)	0.024 ^2^
Self-reported stress symptoms during postpartum period, N (%)	13 (43.3)	11 (68.8)	
Treatment with medication during antenatal period, N (%)		8 (50.0)	<0.001 ^2,^*
Period of medication, N (%)			
1st trimester of pregnancy		2 (12.5)	
3rd trimester of pregnancy		1 (6.3)	
1st and 2nd trimester of pregnancy		1 (6.3)	
2nd and 3rd trimester of pregnancy			
Postpartum (without breastfeeding)		4 (25.0)	
Concerns about medication intake, N (%)		4 (25.0)	
Vaginal delivery, N (%)	18 (60.0)	9 (56.3)	0.806 ^3^
Mild complications of labor and delivery, N (%)	3 (10.0)	4 (25.0)	0.216 ^2^
Support from a healthcare professional/family, N (%)	16 (53.3)	13 (86.7)	0.028 ^3^
Illicit/addictive substances, N (%)			
Alcohol/illegal drugs			
Smoking	10 (33.3)	9 (56.3)	0.133 ^3^
Breastfeeding, N (%)	29 (96.7)	12 (75.0)	0.043 ^2,^*
Duration, mean (SD); weeks	39.8 (32.6)	12.3 (17.8)	0.003 ^4,^*

* Value is significant at *p <* 0.05; ^1^ Student’s *t*-test; ^2^ Fisher’s exact test; ^3^ Pearson’s correlation; ^4^ Mann–Whitney U test; BMI: body mass index; MSc: master of science; PhD: doctor of philosophy.

**Table 2:** Children’s characteristics (N = 46).

		Control Group (Ν = 30)	Children of Mothers with Perinatal Mental Disorders (Ν = 16)	*p*
**Age, Mean (SD): Months**		31.1 (8)	34.9 (8.8)	0.146 ^1^
Sex, Ν (%)	Male	16 (53.3)	8 (50.0)	0.829 ^2^
	Female	14 (46.7)	8 (50.0)	
Birth weight, mean (SD): grams		3205.2 (346.7)	3386.3 (319.2)	0.092 ^1^
Gestational age, mean (SD): weeks		38.7 (1)	38.7 (0.9)	0.947 ^1^
Length at birth, mean (SD): cm		49.4 (3.6)	52.2 (1.7)	0.025 ^1,^*
Head circumference at birth, mean (SD): cm		35.3 (3.5)	34.9 (1.9)	0.736 ^1^
Apgar score, mean (SD)		9 (0)	9 (0)	
Current body weight, mean (SD): kg		13.9 (2.3)	14.5 (2.6)	0.431 ^1^
Current height, mean (SD): cm		93.1 (7.2)	96.2 (6.2)	0.152 ^1^
Current BMI, mean (SD)		16.1 (1.5)	15.6 (1.8)	0.399 ^1^
Current head circumference, mean (SD): cm		50.7 (4.9)	50.1 (1.2)	0.633 ^1^

* Value is significant at *p <* 0.05; ^1^ Student’s *t*-test; ^2^ Pearson’s correlation; BMI: body mass index.

**Table 3:** Correlation among cortisol levels and child behavior check list dimensions.

	Maternal Cortisol		Child’s Cortisol	
	r	*p* ^1^	r	*p* ^1^
Total sample				
Emotionally Reactive	−0.03	0.879	−0.03	0.854
Anxious/Depressed	0.12	0.469	0.05	0.755
Somatic Complaints	−0.04	0.828	−0.04	0.823
Withdrawn	−0.14	0.415	−0.12	0.467
Sleep Problems	−0.14	0.402	−0.06	0.721
Attention Problems	0.09	0.583	−0.07	0.670
Aggressive Behavior	0.18	0.276	0.07	0.695
Internalizing problems	−0.02	0.925	−0.02	0.910
Externalizing problems	0.17	0.316	0.01	0.943
Total problems	0.06	0.722	−0.04	0.792
Affective Problems	−0.03	0.849	0.06	0.741
Anxiety Problems	0.05	0.774	−0.06	0.741
Pervasive developmental problems	−0.07	0.680	−0.11	0.507
Attention Deficit/Hyperactivity Problems	0.00	0.991	−0.12	0.458
Oppositional Defiant Problems	0.26	0.116	0.12	0.486
Control group				
Emotionally Reactive	−0.11	0.623	−0.17	0.411
Anxious/Depressed	0.03	0.888	−0.14	0.501
Somatic Complaints	0.03	0.905	−0.03	0.883
Withdrawn	−0.12	0.588	−0.19	0.375
Sleep Problems	−0.09	0.660	−0.18	0.395
Attention Problems	0.15	0.488	−0.08	0.700
Aggressive Behavior	−0.02	0.914	−0.12	0.571
Internalizing problems	0.03	0.899	−0.10	0.646
Externalizing problems	0.12	0.586	−0.04	0.868
Total problems	0.06	0.795	−0.11	0.602
Affective Problems	0.12	0.572	0.01	0.960
Anxiety Problems	−0.03	0.877	−0.28	0.169
Pervasive developmental problems	−0.02	0.925	−0.15	0.466
Attention Deficit/Hyperactivity Problems	−0.07	0.755	−0.10	0.632
Oppositional Defiant Problems	0.04	0.835	−0.09	0.661
Mothers with perinatal mental disorders				
Emotionally Reactive	0.13	0.679	0.46	0.111
Anxious/Depressed	0.29	0.328	0.57	0.042 *
Somatic Complaints	−0.23	0.456	−0.09	0.770
Withdrawn	−0.17	0.570	−0.05	0.874
Sleep Problems	−0.22	0.468	0.32	0.286
Attention Problems	0.02	0.939	0.04	0.884
Aggressive Behavior	0.61	0.028 *	0.67	0.013 *
Internalizing problems	−0.11	0.710	0.25	0.412
Externalizing problems	0.46	0.115	0.44	0.129
Total problems	0.11	0.725	0.30	0.321
Affective Problems	−0.36	0.233	0.19	0.529
Anxiety Problems	0.18	0.560	0.40	0.181
Pervasive developmental problems	−0.19	0.540	−0.04	0.891
Attention Deficit/Hyperactivity Problems	0.22	0.474	−0.12	0.691
Oppositional Defiant Problems	0.58	0.039 *	0.58	0.037 *

* Value is significant at *p <* 0.05; ^1^ Pearson’s correlation.

## Data Availability

Not applicable.

## References

[1] Duthie L., Reynolds R.M. (2013). Changes in the maternal hypothalamic-pituitary-adrenal axis in pregnancy and postpartum: Influences on maternal and fetal outcomes. Neuroendocrinology.

[2] de Rezende M.G., Garcia-Leal C., de Figueiredo F.P., Cavalli R., Spanghero M.S., Barbieri M.A., Bettiol H., de Castro M., Del-Ben C.M. (2016). Altered functioning of the HPA axis in depressed postpartum women. J. Affect. Disord..

[3] Pragst F., Balikova M.A. (2006). State of the art in hair analysis for detection of drug and alcohol abuse. Clin. Chim. Acta.

[4] Gentile S. (2017). Untreated depression during pregnancy: Short- and long-term effects in offspring. A systematic review. Neuroscience.

[5] Davis E.P., Glynn L.M., Waffarn F., Sandman C.A. (2011). Prenatal maternal stress programs infant stress regulation. J. Child Psychol. Psychiatry.

[6] Bryson H.E., Mensah F., Goldfeld S., Price A.M.H., Giallo R. (2021). Hair cortisol in mother-child dyads: Examining the roles of maternal parenting and stress in the context of early childhood adversity. Eur. Child Adolesc. Psychiatry.

[7] Karlén J., Frostell A., Theodorsson E., Faresjö T., Ludvigsson J. (2013). Maternal influence on child HPA axis: A prospective study of cortisol levels in hair. Pediatrics.

[8] Lembcke H., Buchmüller T., Leyendecker B. (2020). Refugee mother-child dyads’ hair cortisol, post-traumatic stress, and affectionate parenting. Psychoneuroendocrinology.

[9] Liu C.H., Snidman N., Leonard A., Meyer J., Tronick E. (2016). Intra-individual stability and developmental change in hair cortisol among postpartum mothers and infants: Implications for understanding chronic stress. Dev. Psychobiol..

[10] Flom M., St John A.M., Meyer J.S., Tarullo A.R. (2017). Infant hair cortisol: Associations with salivary cortisol and environmental context. Dev. Psychobiol..

[11] Stein A., Pearson R.M., Goodman S.H., Rapa E., Rahman A., McCallum M., Howard L.M., Pariante C.M. (2014). Effects of perinatal mental disorders on the fetus and child. Lancet.

[12] Alink L.R., van Ijzendoorn M.H., Bakermans-Kranenburg M.J., Mesman J., Juffer F., Koot H.M. (2008). Cortisol and externalizing behavior in children and adolescents: Mixed meta-analytic evidence for the inverse relation of basal cortisol and cortisol reactivity with externalizing behavior. Dev. Psychobiol..

[13] Palmer F.B., Anand K.J., Graff J.C., Murphy L.E., Qu Y., Völgyi E., Rovnaghi C.R., Moore A., Tran Q.T., Tylavsky F.A. (2013). Early adversity, socioemotional development, and stress in urban 1-year-old children. J. Pediatr..

[14] Velders F.P., Dieleman G., Henrichs J., Jaddoe V.W., Hofman A., Verhulst F.C., Hudziak J.J., Tiemeier H. (2011). Prenatal and postnatal psychological symptoms of parents and family functioning: The impact on child emotional and behavioural problems. Eur. Child Adolesc. Psychiatry.

[15] Madianos M., Papaghelis M., Philippakis A. (1997). The reliability of SCID I in Greece in clinical and general population. Psychiatriki.

[16] Lyrakos G.N., Arvaniti C., Smyrnioti M., Kostopanagiotou G. (2011). Translation and validation study of the depression anxiety stress scale in the Greek general population and in a psychiatric patient’s sample. Eur. Psychiatry.

[17] Lovibond S.H., Lovibond P.F. (1995). Manual for the Depression Anxiety Stress Scales.

[18] Knobloch H., Stevens F.M., Malone A.F. (1980). Manual of Developmental Diagnosis. The Administration and Interpretation of the Revised Gesell and Amtruda Developmental and Neurologic Examination.

[19] Zimmerman I.L., Steiner V.G., Pond R.E. (2011). Preschool Language Scales.

[20] Tsaousis I. (2009). Checklist for Problematic Behavior 1½-5.

[21] Achenbach T.M. (2009). The Achenbach System of Empirically Based Assessment (ASEBA): Development, Findings, Theory, and Applications.

[22] Laplante D.P., Barr R.G., Brunet A., Galbaud du Fort G., Meaney M.L., Saucier J.F., Zelazo P.R., King S. (2004). Stress during pregnancy affects general intellectual and language functioning in human toddlers. Pediatr. Res..

[23] Laplante D., Brunet A., Schmitz N., Ciampi A., King S. (2008). Project Ice Storm: Prenatal Maternal Stress Affects Cognitive and Linguistic Functioning in 5½-Year-Old Children. J. Am. Acad. Child Adolesc. Psychiatry.

[24] Anderson J.W., Johnstone B.M., Remley D.T. (1999). Breast-feeding and cognitive development: A meta-analysis. Am. J. Clin. Nutr..

[25] Kim K.M., Choi J.W. (2020). Associations between breastfeeding and cognitive function in children from early childhood to school age: A prospective birth cohort study. Int. Breastfeed J..

[26] Skurtveit S., Selmer R., Roth C., Hernandez-Diaz S., Handal M. (2014). Prenatal exposure to antidepressants and language competence at age three: Results from a large population-based pregnancy cohort in Norway. BJOG.

[27] Dawson G., Ashman S.B., Carver L.J. (2000). The role of early experience in shaping behavioral and brain development and its implications for social policy. Dev. Psychopathol..

[28] Van Batenburg-Eddes T., Brion M.J., Henrichs J., Jaddoe V.W., Hofman A., Verhulst F.C., Lawlor D.A., Davey Smith G., Tiemeier H. (2013). Parental depressive and anxiety symptoms during pregnancy and attention problems in children: A cross-cohort consistency study. J. Child Psychol. Psychiatry.

[29] Schneider M.L., Coe C.L. (1993). Repeated social stress during pregnancy impairs neuromotor development of the primate infant. J. Dev. Behav. Pediatr..

[30] Liu C.H., Fink G., Brentani H., Brentani A. (2017). An assessment of hair cortisol among postpartum Brazilian mothers and infants from a high-risk community in São Paulo: Intra-individual stability and association in mother-infant dyads. Dev. Psychobiol..

[31] Broeks C.W., Choenni V., Kok R., van der Voorn B., de Kruijff I., van den Akker E., van Rossum E., Hoogendijk W., Hillegers M., Kamperman A.M. (2021). An exploratory study of perinatal hair cortisol concentrations in mother-infant dyads with severe psychiatric disorders versus healthy controls. BJPsych Open.

[32] Galbally M., van Rossum E., Watson S.J., de Kloet E.R., Lewis A.J. (2019). Trans-generational stress regulation: Mother-infant cortisol and maternal mental health across the perinatal period. Psychoneuroendocrinology.

[33] Gunnar M., Talge M.N., Schmidt L.A., Segalowitz S.J. (2008). Neuroendocrine measures in developmental research. Developmental Psychophysiology: Theory, Systems, and Methods.

[34] Ouellette S.J., Russell E., Kryski K.R., Sheikh H.I., Singh S.M., Koren G., Hayden E.P. (2015). Hair cortisol concentrations in higher- and lower-stress mother-daughter dyads: A pilot study of associations and moderators. Dev. Psychobiol..

[35] Weinstock M. (2005). The potential influence of maternal stress hormones on development and mental health of the offspring. Brain Behav. Immun..

[36] Gjerde L.C., Eilertsen E.M., Reichborn-Kjennerud T., McAdams T.A., Zachrisson H.D., Zambrana I.M., Røysamb E., Kendler K.S., Ystrom E. (2017). Maternal perinatal and concurrent depressive symptoms and child behavior problems: A sibling comparison study. J. Child Psychol. Psychiatry.

[37] Hoffman M.C., Karban L.V., Benitez P., Goodteacher A., Laudenslager M.L. (2014). Chemical processing and shampooing impact cortisol measured in human hair. Clin. Investig. Med..

[38] Wosu A.C., Valdimarsdóttir U., Shields A.E., Williams D.R., Williams M.A. (2013). Correlates of cortisol in human hair: Implications for epidemiologic studies on health effects of chronic stress. Ann. Epidemiol..

